# Synthesis and structure of {methyl (*Z*)-2-[4-(di­meth­yl­amino)­benzyl­idene]hydrazine-1-carbodi­thio­ate-κ^2^*N*^2^,*S*}bis­(tri­phenyl­phosphine-κ*P*)copper(I) nitrate carbon tetra­chloride monosolvate

**DOI:** 10.1107/S2056989026000228

**Published:** 2026-01-16

**Authors:** Liji Muthirakalayil Abraham, Banmankhraw Dkhar, Amirthalingam Arunkumar, Thangaraja Chinnathangavel, Kolandaivelu Saminathan, Marappan Velusamy, Venugopal Rajendiran

**Affiliations:** a Department of Chemistry School of Basic and Applied Sciences Central University of Tamil Nadu Thiruvarur - 610 005 India; b Department of Chemistry North Eastern Hill University,Shillong 793022 India; c Department of Chemistry, Anna University Regional Campus, Madurai 625019, India; d Department of Physics, Anna University/University VOC College of Engineering, Tuticorin Campus, Tuticorin 628802, India; University of Aberdeen, United Kingdom

**Keywords:** copper(I), tri­phenyl­phosphine, distorted tetra­hedral geometry, crystal structure

## Abstract

In the title compound, [Cu(C_11_H_15_N_3_S_2_){P(C_6_H_5_)_3_}_2_]NO_3_·CCl_4_, the carbon tetra­chloride solvent mol­ecule is presumed to have originated as an impurity in the chloro­form solvent used. The coordination environment around the copper(I) ion is a distorted CuNSP_2_ tetra­hedron, with a τ_4_ structural index of 0.844. In the extended structure, the cation and anion are linked by an N—H⋯O hydrogen bond. Along with electrostatic forces, C—H⋯N, C—H⋯S and C—H⋯O hydrogen bonds help to consolidate the crystal packing.

## Abstract 2

In the title compound, the carbon tetra­chloride solvent mol­ecule is presumed to have originated as an impurity in the chloro­form solvent used. In the extended structure, the cation and anion are linked by an N—H⋯O hydrogen bond. Along with electrostatic forces, C—H⋯N, C—H⋯S and C—H⋯O hydrogen bonds help to consolidate the crystal packing.

## 1. Chemical context

Copper(I) complexes exhibit broad applications across medicinal chemistry (Papazoglou *et al.*, 2014 ▸), materials science (Hei & Li, 2021 ▸), and catalysis (Egbert *et al.*, 2013 ▸). Elucidating their structural features provides valuable insights for the innovative design of further copper(I) complexes, thereby enhancing their structure–activity relationships.
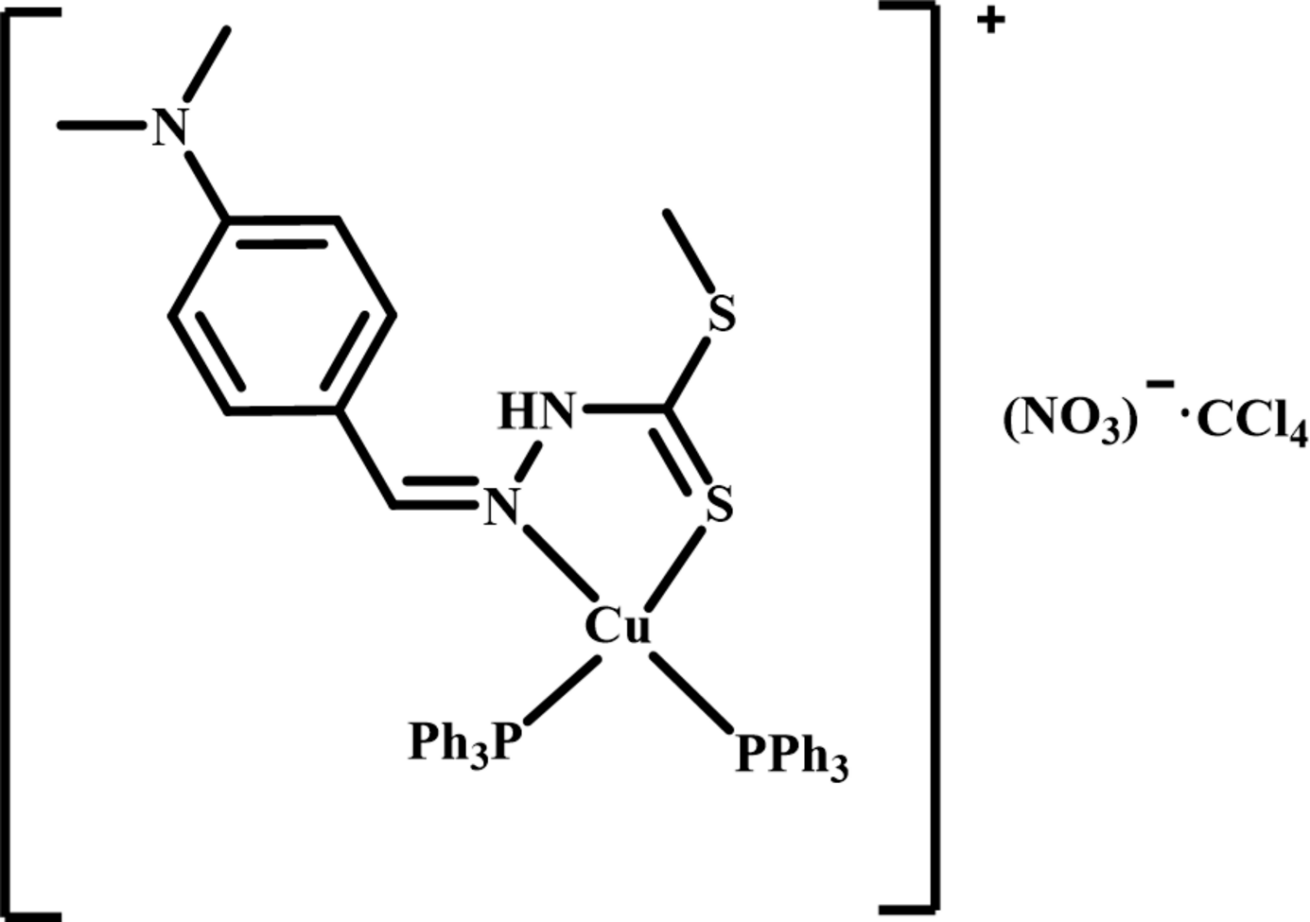


Copper(I) complexes bearing nitro­gen and sulfur donor ligands are of significant inter­est owing to the presence of this metal in the active sites of hydrogenases, carbon monoxide de­hydrogenases, and blue copper proteins. Complexes of copper(I) with methyl (*Z*)-2-(4-(di­methyl­amino)­benzyl­idene)hydrazine-1-carbodi­thio­ate ligands and their BSA binding properties have been reported in the literature (Malakar *et al.*, 2023 ▸). Such copper(I) species are generally obtained by reacting methyl (*Z*)-2-(4-(di­methyl­amino)­benzyl­idene)hydrazine-1-carbodi­thio­ate or its derivatives with appropriate copper(I) precursors. In this context, the present work reports the synthesis and single-crystal X-ray characterization of the title mononuclear mixed-ligand copper(I) complex [Cu(C_11_H_15_N_3_S_2_){P(C_6_H_5_)_3_}_2_]NO_3_·CCl_4_ (**I**) or [Cu(*NS*)(PPh_3_)_2_]NO_3_·CCl_4_, where *NS* denotes the methyl (*Z*)-2-(4-(di­methyl­amino)­benzyl­idene)hydrazine-1-carbodi­thio­ate chel­ating ligand and PPh_3_ represents the tri­phenyl­phosphine co-ligand.

## 2. Structural commentary

**Figure 1:**
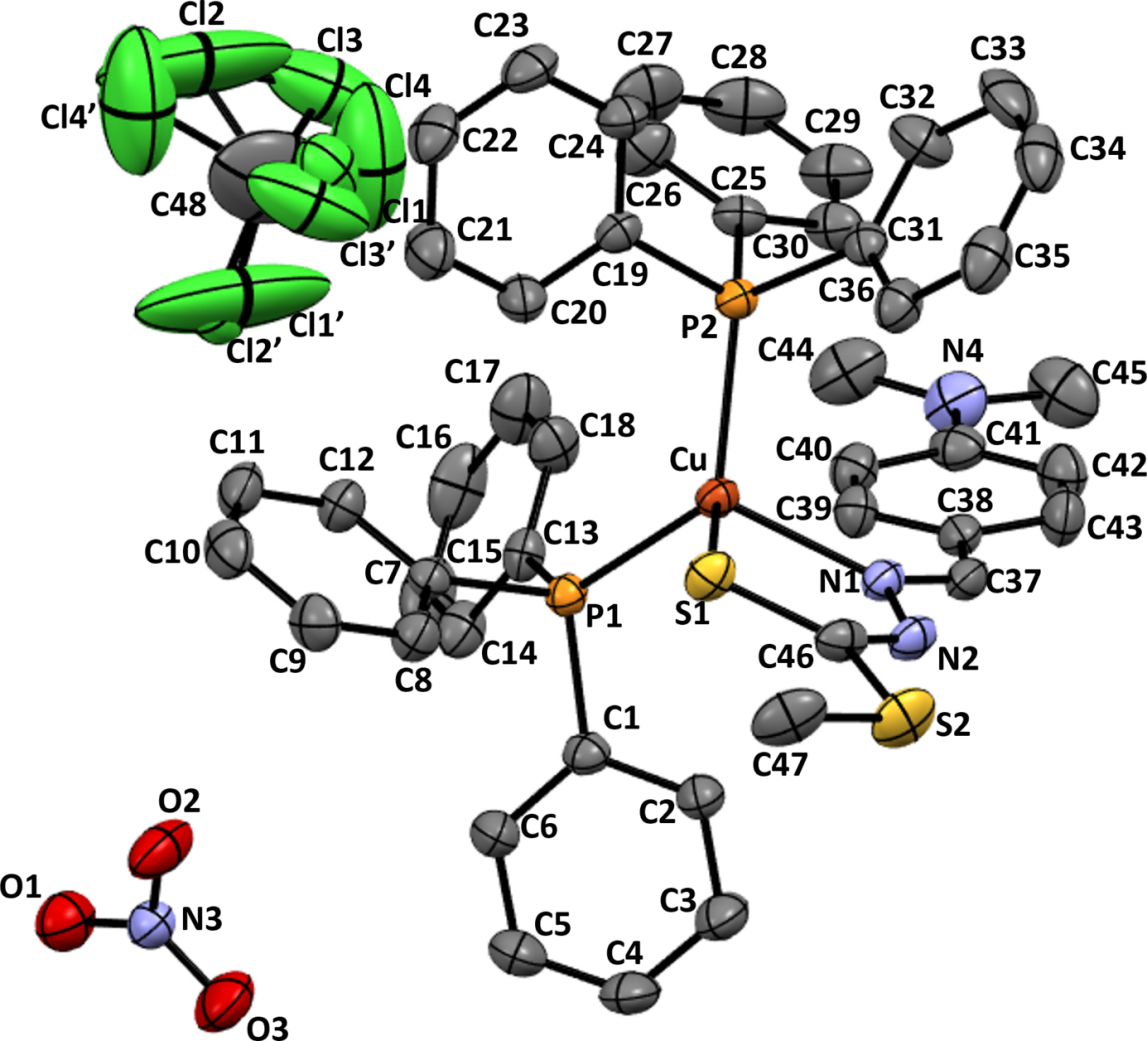
The mol­ecular structure of (**I**) with displacement ellipsoids drawn at the 30% probability level. H atoms are omitted for clarity.

Compound (**I**) crystallizes in the monoclinic space group *P*2_1_/*n*, with one cation, one anion and one disordered CCl_4_ solvent mol­ecule in the asymmetric unit (Fig. 1 ▸). The Cu^I^ atom is bound to an azomethine nitro­gen atom and a sulfur atom from the C_11_H_15_N_3_S_2_ ligand, generating a five-membered chelate ring, in addition to phospho­rous coordination from two tri­phenyl­phosphine ligands. The Cu—N1 bond distance [2.112 (3) Å] is substanti­ally shorter than the Cu—S1 distance [2.3599 (11) Å], which is consistent with previously reported complexes containing analogous donor sets (Malakar *et al.*, 2023 ▸). The bite angle of the di­thio­carbazate fragment, N1—Cu—S1 [84.80 (10)°], is significantly narrower than the bond angles involving the phosphine donors, P1—Cu—P2 [125.29 (4)°], reflecting trends observed in related Cu^I^ systems incorporating such coordination motifs (Pathaw *et al.*, 2021 ▸). The remaining angles in the coordination sphere, namely N1—Cu—P2 [110.95 (9)°], N1—Cu—P1 [115.78 (8)°], S1—Cu—P2 [109.19 (3)°], and S1—Cu—P1 [101.91 (4)°] lie between these two extremes, reflecting the geometric adjustments required to accommodate different donor atoms and steric constraints around the Cu^I^ centre. The notably wider P1—Cu—P2 bond angle can be ascribed to the steric bulk and spatial demands of the two tri­phenyl­phosphine ligands, as observed in Cu^I^ complexes containing similar types of phosphine ligands (Messmer & Palenik, 2011 ▸). The bite angle of the N2—Cu1—S1 chelate ring is intrinsically constrained by the five-membered di­thio­carbazate ring, forcing a much smaller angle than the ideal tetra­hedral value. Overall, these angular distortions are a direct consequence of the competing electronic and steric influences within the coordination sphere, leading to the observed deviation from perfect tetra­hedral geometry. This is reflected in the four-coordinate structural index (τ_4_) of 0.844 [τ_4_ = (360° – (α + β))/141°] where α and β represent the two predominant θ angles in the four-coordinate complex (Yang *et al.*, 2007 ▸): τ_4_ is unity and zero for perfect tetra­hedral and square planar geometries, respectively).

Even though (**I**) was synthesized using chloro­form, the single-crystal X-ray structure revealed the presence of included carbon tetra­chloride (CCl_4_) mol­ecules. This can arise as commercial chloro­form often contains trace amounts of CCl_4_ as a stabilizer or residual impurity from industrial production. These minor amounts of solvate can crystallize during slow evaporation and be incorporated in the crystal. As a result of the weak van der Waals inter­actions, the CCl_4_ mol­ecules occupy voids in the crystal rather than coordinating to the metal centre (Huber *et al.*, 1978 ▸).

## 3. Supra­molecular features

**Figure 2:**
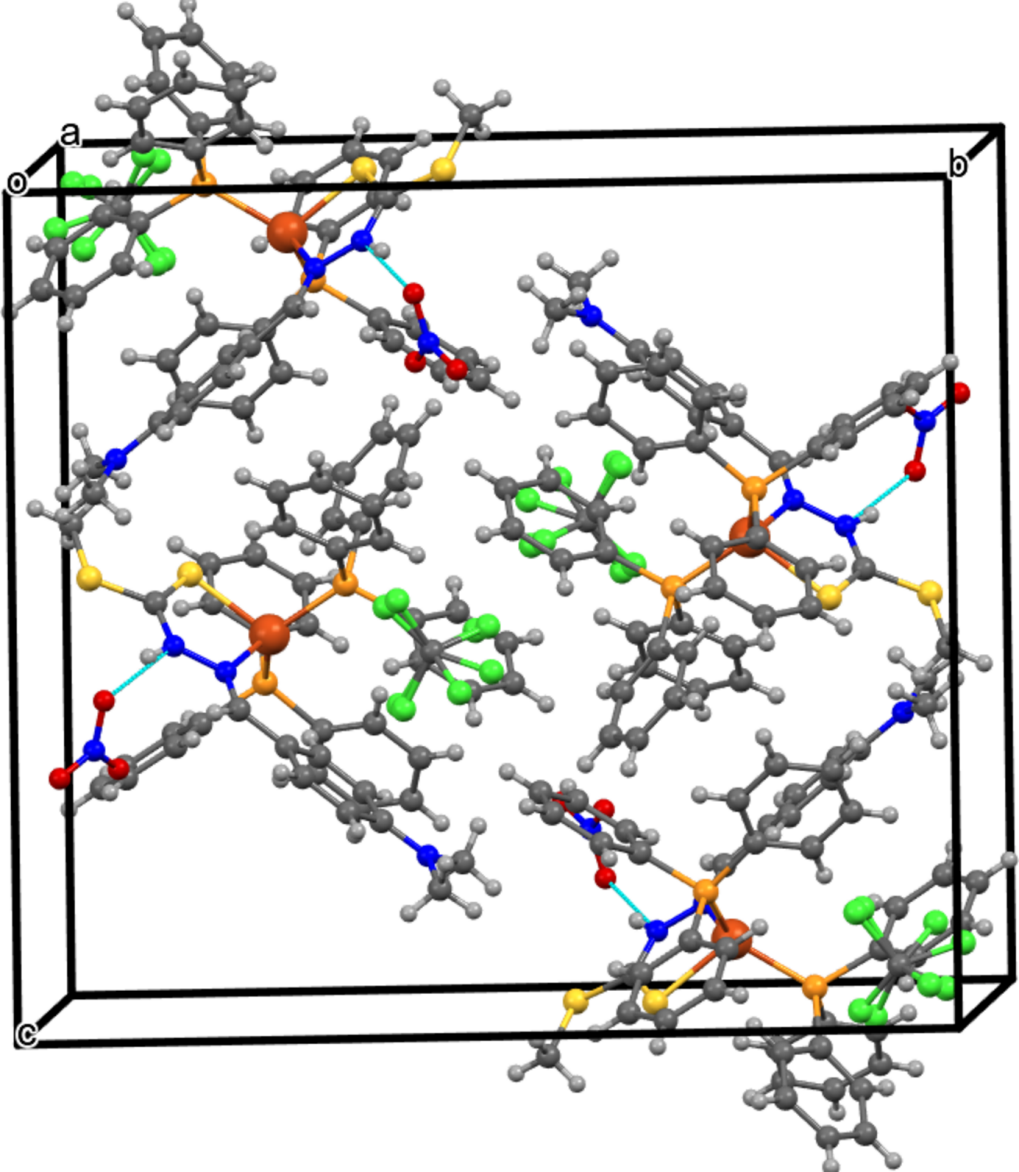
The crystal packing of (**I**) with the N—H⋯O hydrogen bonds shown as blue dashed lines.

In the crystal, an N—H⋯O bond (Table 1 ▸) links the cation with the anion. The packing in the extended structure of (**I**) is consolidated by C—H⋯N, C—H⋯S and C—H⋯O inter­actions. All of the hydrogen-to-acceptor distances are less than 2.9 Å, and the donor-to-acceptor distances are less than 3.5 Å. Moreover, all of the hydrogen-bonding inter­actions exhibit *D*—H⋯*A* bond angles greater than 130°. The complete inter­action details are illustrated in the packing diagram of the compound shown in Fig. 2 ▸.

## 4. Hirshfeld Surface Analysis

**Figure 3:**
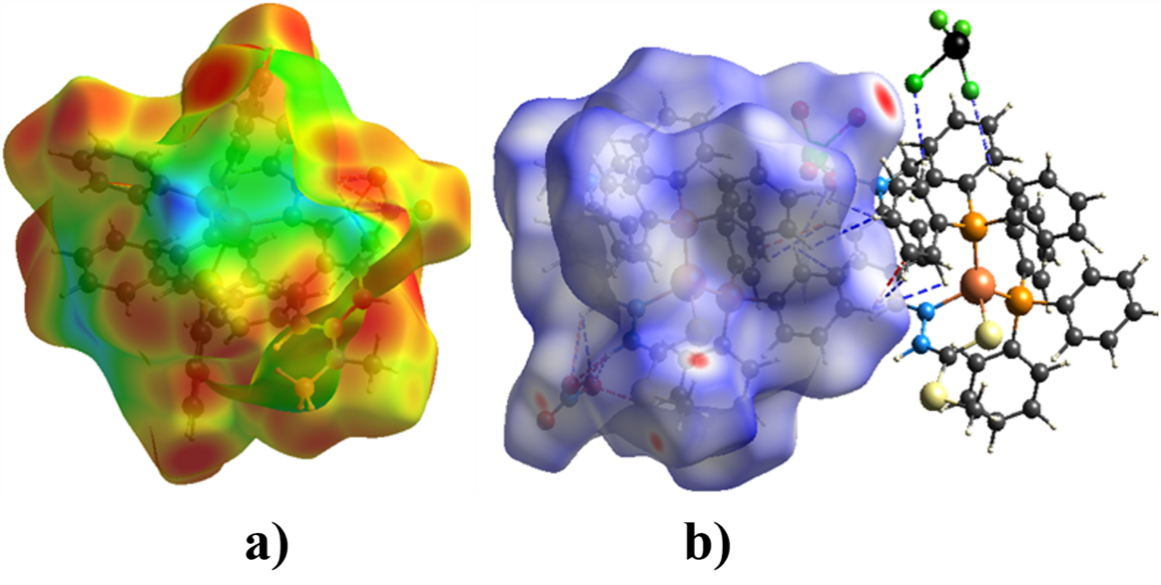
Hirshfeld surfaces for (**I**); (*a*) *d*_i_ plot; (*b*) *d_norm_* plot.

Hirshfeld surface analysis was carried out using the *Crystal Explorer 21.5* software package. The surfaces were generated over *d*_norm_ and two-dimensional fingerprint plots were obtained to qu­antify the directional inter­molecular inter­actions along with other atom-to-atom contacts. Fig. 3 ▸(*a*) and (*b*) show the Hirshfeld surfaces mapped over *d*_i_ and *d*_norm_, respectively. The dark-red spots indicate the presence of close contacts between atoms, while the green regions represent weak contacts. The blue regions, which occupy the majority of the surface, indicate the absence of close contacts in the structure. In Fig. 3 ▸(*b*), hydrogen-bond inter­actions are represented by red dotted lines, whereas other atom-to-atom inter­actions are represented by blue dotted lines.

**Figure 4:**
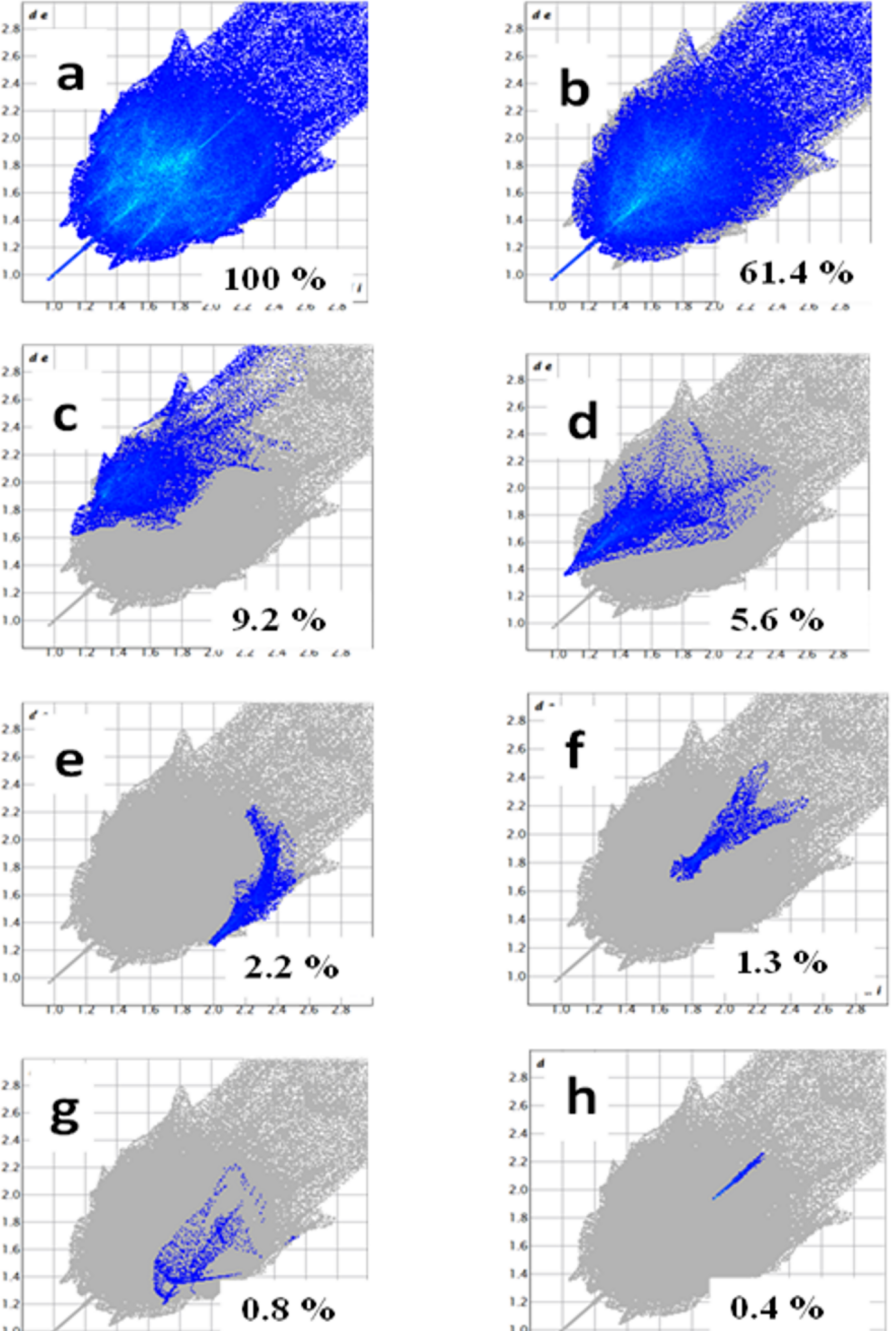
Two-dimensional fingerprint plots for (**I**); (*a*) all inter­actions and delineated into (*b*) H⋯H; (*c*) C⋯H/H⋯C; (*d*) O⋯H/H⋯O; (*e*) S⋯H/H⋯S; (*f*) C⋯C; (*g*) N⋯H/H⋯N; (*h*) S⋯S.

According to the two-dimensional fingerprint plots for (**I**) (Fig. 4 ▸), the H⋯H contacts make the largest contribution (61.4%) to the total Hirshfeld surface at a distance range of *d*_e_ + *d*_i_ ≃ 1.9 Å. Similarly, the C⋯H, O⋯H, S⋯H, C⋯C, N⋯H, and S⋯S inter­actions contribute 9.2%, 5.6%, 2.2%, 1.3%, 0.8%, and 0.4%, respectively.

## 5. Database survey

A SciFinder structure-similarity search for Cu^I^ complexes bearing *N,S*-bidentate hydrazine-derived carbodi­thio­ate ligands in combination with tri­phenyl­phosphine donors revealed a small but significant group of structurally related systems. Early studies by Bianchini and co-workers explored the reactivity of bis­(tri­phenyl­phosphine)copper(I) species toward heterocumulenes such as CO_2_, COS, CS_2_, and phenyl iso­thio­cyanate, establishing that Cu^I^ centres supported by phosphines and sulfur-bearing ligands favour distorted tetra­hedral coordination and readily engage in S-based bond formation. The Cu—S distances vary from 2.10–2.35 Å for monodentate thiol­ates to 2.40–2.48 Å in chelating di­thiol­ate environments, while the Cu—P distances lie near 2.27 Å (Bianchini *et al.*, 1983 ▸, 2002*a* ▸,*b* ▸). Borate-anchored Cu^I^–phosphine complexes were reported by Lobbia *et al.* (1997 ▸) in which the Cu atom is coordinated to one phosphine and three pyrazolyl nitro­gen atoms in a distorted tetra­hedral environment. The N—Cu—P angles fall in the range 120.8 (1)–130.3 (1)°, and the N—Cu—N angles between 87.5 (1) and 90.8 (1)°, indicating that steric effects from the bulky PCy_3_ ligand significantly influence the coordination geometry. Complementary insight into the structural variability of phosphine–supported Cu(I) environments was provided by Bowmaker *et al.* (2002 ▸), who characterised three-coordinate tri­cyclo­hexyl­phosphine complexes, which crystallise in several polymorphic forms but maintain Cu—P distances in the 2.20–2.29 Å range and exhibit comparable P—Cu—P angles, and acyl­pyrazolo­nate bis­(phosphine) derivatives were described by Marchetti *et al.*, (2000 ▸) and Eller & Kubas, (2002 ▸), who demonstrated that sulfur dioxide binding to Cu^I^ phosphine thiol­ate systems stabilizes unusual S- and Se-coordinated adducts, which further expanded the structural space, confirming that phosphine steric effects and ancillary ligand denticity modulate tetra­hedral *versus* pseudo-trigonal coordination. The adaptability of Cu^I^ coordination spheres in the presence of mixed N- and S-donors was additionally illustrated in phenanthroline-containing systems (Mutrofin *et al.*, 2008 ▸; Pettinari *et al.*, 1996 ▸) who reported phosphine-stabilized Cu^I^–pyrazole salts that display diverse supra­molecular assemblies through hydrogen-bonding inter­actions. Across this literature landscape, *κ^2^-N,S* chelation in combination with monodentate phosphine donors emerges as a recurring theme. Several copper(I) and copper(II) systems with tri­cyclo­hexyl- or tri­phenyl­phosphine donors were reported, as well as analogous Ni, Pd, Pt, Ag, and Ru complexes. Notably, nitrate-bound tri­cyclo­hexyl­phosphine copper complexes and thiol­ate-bridged Cu^I^–phosphine derivatives exhibit similar coordination features. However, no previous report describes a Cu^I^ system incorporating a methyl-substituted (*Z*)-hydrazine-1-carbodi­thio­ate ligand combined with tri­phenyl­phosphine and nitrate, confirming the novelty of the present structure.

## 6. Synthesis and crystallization

To a 20 ml chloro­form solution of the metal precursor [Cu(PPh_3_)_2_NO_3_] (0.325 g, 0.500 mmol), the ligand methyl (*Z*)-2-(4-(di­methyl­amino)­benzyl­idene)hydrazine-1-carbodi­thio­ate (0.126 g, 0.500 mmol) was added and stirred at room temperature for 12 h. The solution was then evaporated, and the desired complex was precipitated by diethyl ether (40 ml) and dried under vacuum. The obtained product was then recrystallized from chloro­form solution by slow evaporation to give yellow needles of (**I**). Yield: 65%.

## 7. Refinement

Crystal data, data collection and structure refinement details are summarized in Table 2 ▸. H atoms were positioned geometrically (C—H = 0.93–0.96 Å) and refined as riding with *U*_iso_(H) = 1.2–1.5*U*_eq_(C).

## Supplementary Material

Crystal structure: contains datablock(s) I. DOI: 10.1107/S2056989026000228/hb8181sup1.cif

Structure factors: contains datablock(s) I. DOI: 10.1107/S2056989026000228/hb8181Isup2.hkl

CCDC reference: 2482415

Additional supporting information:  crystallographic information; 3D view; checkCIF report

## Figures and Tables

**Table 1:** Hydrogen-bond geometry (Å, °)

*D*—H⋯*A*	*D*—H	H⋯*A*	*D*⋯*A*	*D*—H⋯*A*
N2—H2*A*⋯O2^i^	0.82	1.96	2.754 (5)	163
C2—H2⋯N1	0.93	2.55	3.391 (5)	151
C36—H36⋯S1	0.93	2.87	3.758 (5)	160
C37—H37⋯O2^i^	0.93	2.66	3.384 (5)	135
C47—H47*A*⋯O2	0.96	2.52	3.322 (6)	141

Symmetry code: (i) 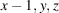.

**Table 2:** Experimental details

Crystal data	
Chemical formula	[Cu(C_11_H_15_N_3_S_2_)(C_18_H_15_P)_2_]NO_3_·CCl_4_
*M*_r_	1057.28
Crystal system, space group	Monoclinic, *P*2_1_/*n*
Temperature (K)	293
*a*, *b*, *c* (Å)	10.8364 (18), 22.358 (4), 20.107 (4)
β (°)	98.146 (7)
*V* (Å^3^)	4822.4 (16)
*Z*	4
Radiation type	Mo *K*α
μ (mm^−1^)	0.87
Crystal size (mm)	0.30 × 0.22 × 0.20

Data collection	
Diffractometer	Bruker D8 VENTURE CCD
Absorption correction	Multi-scan (*SADABS*; Krause et al., 2015 ▸)
*T*_min_, *T*_max_	0.780, 0.845
No. of measured, independent and observed [*I* > 2σ(*I*)] reflections	85779, 8478, 6143
*R*_int_	0.097
(sin θ/λ)_max_ (Å^−1^)	0.595

Refinement	
*R*[*F*^2^ > 2σ(*F*^2^)], *wR*(*F*^2^), *S*	0.055, 0.168, 1.04
No. of reflections	8478
No. of parameters	590
No. of restraints	52
H-atom treatment	H-atom parameters constrained
Δρ_max_, Δρ_min_ (e Å^−3^)	0.57, −0.91

Computer programs: *APEX3* and *SAINT* ▸), *SHELXT2018/2* (Sheldrick, 2015*a* ▸), *SHELXL2019/2* (Sheldrick, 2015*b* ▸), *Mercury* (Macrae *et al.*, 2020 ▸) and *publCIF*(Westrip, 2010 ▸).

## supplementary crystallographic information

### {Methyl (Z)-2-[4-(dimethylamino)benzylidene]hydrazine-1-carbodithioate-κ2N2,S}bis(triphenylphosphine-κP)copper(I) nitrate carbon tetrachloride monosolvate Crystal data

**Table:**

[Cu(C_11_H_15_N_3_S_2_)(C_18_H_15_P)_2_]NO_3_·CCl_4_	*F*(000) = 2176
*M**_r_* = 1057.28	*D*_x_ = 1.456 Mg m^−^^3^
Monoclinic, *P*2_1_/*n*	Mo *K*α radiation, λ = 0.71073 Å
*a* = 10.8364 (18) Å	Cell parameters from 8478 reflections
*b* = 22.358 (4) Å	θ = 2.0–25.0°
*c* = 20.107 (4) Å	µ = 0.87 mm^−^^1^
β = 98.146 (7)°	*T* = 293 K
*V* = 4822.4 (16) Å^3^	Needle, yellow
*Z* = 4	0.30 × 0.22 × 0.20 mm

### {Methyl (Z)-2-[4-(dimethylamino)benzylidene]hydrazine-1-carbodithioate-κ2N2,S}bis(triphenylphosphine-κP)copper(I) nitrate carbon tetrachloride monosolvate Data collection

**Table:**

Bruker D8 VENTURE CCD diffractometer	6143 reflections with *I* > 2σ(*I*)
ω scans	*R*_int_ = 0.097
Absorption correction: multi-scan (SADABS; Krause et al., 2015)	θ_max_ = 25.0°, θ_min_ = 2.0°
*T*_min_ = 0.780, *T*_max_ = 0.845	*h* = −12→12
85779 measured reflections	*k* = −26→26
8478 independent reflections	*l* = −23→23

### {Methyl (Z)-2-[4-(dimethylamino)benzylidene]hydrazine-1-carbodithioate-κ2N2,S}bis(triphenylphosphine-κP)copper(I) nitrate carbon tetrachloride monosolvate Refinement

**Table:**

Refinement on *F*^2^	Hydrogen site location: inferred from neighbouring sites
Least-squares matrix: full	H-atom parameters constrained
*R*[*F*^2^ > 2σ(*F*^2^)] = 0.055	*w* = 1/[σ^2^(*F*_o_^2^) + (0.0965*P*)^2^ + 2.9941*P*] where *P* = (*F*_o_^2^ + 2*F*_c_^2^)/3
*wR*(*F*^2^) = 0.168	(Δ/σ)_max_ = 0.002
*S* = 1.04	Δρ_max_ = 0.57 e Å^−^^3^
8478 reflections	Δρ_min_ = −0.91 e Å^−^^3^
590 parameters	Extinction correction: SHELXL-2019/2 (Sheldrick 2015b), Fc^*^=kFc[1+0.001xFc^2^λ^3^/sin(2θ)]^-1/4^
52 restraints	Extinction coefficient: 0.0013 (3)
Primary atom site location: dual	

### {Methyl (Z)-2-[4-(dimethylamino)benzylidene]hydrazine-1-carbodithioate-κ2N2,S}bis(triphenylphosphine-κP)copper(I) nitrate carbon tetrachloride monosolvate Special details

**Table:**

Geometry. All esds (except the esd in the dihedral angle between two l.s. planes) are estimated using the full covariance matrix. The cell esds are taken into account individually in the estimation of esds in distances, angles and torsion angles; correlations between esds in cell parameters are only used when they are defined by crystal symmetry. An approximate (isotropic) treatment of cell esds is used for estimating esds involving l.s. planes.

### {Methyl (Z)-2-[4-(dimethylamino)benzylidene]hydrazine-1-carbodithioate-κ2N2,S}bis(triphenylphosphine-κP)copper(I) nitrate carbon tetrachloride monosolvate Fractional atomic coordinates and isotropic or equivalent isotropic displacement parameters (Å^2^)

**Table:**

	*x*	*y*	*z*	*U*_iso_*/*U*_eq_	Occ. (<1)
C1	3.0510 (3)	0.13767 (17)	0.68718 (18)	0.0465 (9)	
C2	2.9398 (4)	0.1173 (2)	0.7063 (2)	0.0553 (10)	
H2	2.867151	0.139233	0.694544	0.066*	
C3	2.9357 (5)	0.0647 (2)	0.7424 (2)	0.0726 (14)	
H3	2.860989	0.051497	0.754977	0.087*	
C4	3.0431 (5)	0.0325 (2)	0.7595 (3)	0.0788 (15)	
H4	3.040878	−0.002617	0.784103	0.095*	
C5	3.1524 (5)	0.0510 (2)	0.7411 (3)	0.0753 (14)	
H5	3.224366	0.028597	0.753176	0.090*	
C6	3.1578 (4)	0.1033 (2)	0.7043 (2)	0.0628 (12)	
H6	3.232832	0.115400	0.691075	0.075*	
C7	3.1742 (3)	0.19743 (17)	0.58957 (18)	0.0427 (9)	
C8	3.1793 (4)	0.1447 (2)	0.5522 (2)	0.0593 (11)	
H8	3.120031	0.114950	0.554549	0.071*	
C9	3.2713 (4)	0.1363 (2)	0.5120 (2)	0.0665 (12)	
H9	3.275139	0.100776	0.488417	0.080*	
C10	3.3567 (4)	0.1805 (3)	0.5071 (2)	0.0707 (13)	
H10	3.418250	0.174973	0.479791	0.085*	
C11	3.3520 (4)	0.2327 (2)	0.5420 (3)	0.0702 (13)	
H11	3.409354	0.262965	0.537876	0.084*	
C12	3.2604 (4)	0.2407 (2)	0.5842 (2)	0.0565 (11)	
H12	3.258766	0.275928	0.608712	0.068*	
C13	3.1098 (3)	0.26363 (19)	0.70024 (19)	0.0485 (10)	
C14	3.1983 (4)	0.2512 (2)	0.7557 (2)	0.0629 (12)	
H14	3.225773	0.212115	0.763530	0.075*	
C15	3.2451 (5)	0.2949 (3)	0.7986 (3)	0.0864 (17)	
H15	3.305382	0.285752	0.834819	0.104*	
C16	3.2045 (6)	0.3521 (4)	0.7888 (3)	0.098 (2)	
H16	3.236378	0.381759	0.818852	0.118*	
C17	3.1166 (5)	0.3669 (3)	0.7349 (3)	0.0907 (18)	
H17	3.089342	0.406184	0.728159	0.109*	
C18	3.0693 (4)	0.3218 (2)	0.6906 (2)	0.0657 (12)	
H18	3.009593	0.331144	0.654129	0.079*	
C19	2.9079 (3)	0.31867 (17)	0.44612 (18)	0.0440 (9)	
C20	3.0136 (4)	0.2842 (2)	0.4469 (2)	0.0606 (12)	
H20	3.032798	0.255689	0.480405	0.073*	
C21	3.0921 (5)	0.2914 (2)	0.3982 (2)	0.0740 (14)	
H21	3.162242	0.267328	0.398696	0.089*	
C22	3.0651 (5)	0.3343 (2)	0.3496 (2)	0.0718 (13)	
H22	3.118750	0.340393	0.317991	0.086*	
C23	2.9598 (4)	0.3679 (2)	0.3474 (2)	0.0661 (12)	
H23	2.941286	0.396449	0.313889	0.079*	
C24	2.8801 (4)	0.35993 (19)	0.3947 (2)	0.0590 (11)	
H24	2.807351	0.382426	0.392042	0.071*	
C25	2.8157 (4)	0.37910 (17)	0.55577 (19)	0.0492 (10)	
C26	2.9089 (5)	0.4208 (2)	0.5522 (3)	0.0719 (13)	
H26	2.967071	0.414072	0.523193	0.086*	
C27	2.9176 (6)	0.4719 (2)	0.5906 (3)	0.0962 (19)	
H27	2.982037	0.498936	0.587929	0.115*	
C28	2.8308 (7)	0.4832 (3)	0.6330 (3)	0.098 (2)	
H28	2.835634	0.517911	0.658740	0.118*	
C29	2.7377 (6)	0.4428 (2)	0.6367 (3)	0.0832 (16)	
H29	2.678614	0.450020	0.665011	0.100*	
C30	2.7305 (5)	0.3912 (2)	0.5987 (2)	0.0627 (12)	
H30	2.666758	0.363976	0.602099	0.075*	
C31	2.6534 (3)	0.30880 (18)	0.46096 (18)	0.0467 (9)	
C32	2.5847 (5)	0.3598 (2)	0.4473 (3)	0.0778 (15)	
H32	2.610620	0.395616	0.468235	0.093*	
C33	2.4753 (5)	0.3572 (3)	0.4016 (3)	0.0947 (19)	
H33	2.428118	0.391707	0.392422	0.114*	
C34	2.4358 (5)	0.3051 (3)	0.3702 (3)	0.0833 (17)	
H34	2.363814	0.304342	0.338950	0.100*	
C35	2.5023 (5)	0.2553 (3)	0.3849 (2)	0.0790 (15)	
H35	2.474493	0.219539	0.364303	0.095*	
C36	2.6122 (4)	0.2554 (2)	0.4304 (2)	0.0595 (11)	
H36	2.657198	0.220296	0.440074	0.071*	
C37	2.6272 (4)	0.20298 (18)	0.6466 (2)	0.0503 (10)	
H37	2.553203	0.181747	0.645880	0.060*	
C38	2.6472 (3)	0.25264 (19)	0.69251 (19)	0.0491 (10)	
C39	2.7613 (4)	0.28038 (19)	0.7111 (2)	0.0540 (10)	
H39	2.830430	0.266560	0.693121	0.065*	
C40	2.7754 (4)	0.3271 (2)	0.7548 (2)	0.0591 (11)	
H40	2.853898	0.344107	0.766161	0.071*	
C41	2.6747 (4)	0.3504 (2)	0.7831 (2)	0.0616 (11)	
C42	2.5603 (4)	0.3218 (2)	0.7659 (3)	0.0718 (13)	
H42	2.491220	0.335163	0.784262	0.086*	
C43	2.5479 (4)	0.2746 (2)	0.7227 (3)	0.0689 (13)	
H43	2.470375	0.256280	0.712970	0.083*	
C44	2.8032 (6)	0.4290 (3)	0.8405 (3)	0.0986 (19)	
H44A	2.794740	0.461341	0.870926	0.148*	
H44B	2.865745	0.401674	0.860798	0.148*	
H44C	2.827278	0.444588	0.799718	0.148*	
C45	2.5846 (6)	0.4168 (3)	0.8588 (3)	0.1000 (19)	
H45A	2.608369	0.450975	0.886641	0.150*	
H45B	2.514892	0.427070	0.825787	0.150*	
H45C	2.561684	0.384591	0.886145	0.150*	
C46	2.7232 (4)	0.11030 (17)	0.5255 (2)	0.0520 (10)	
C47	2.7519 (6)	0.0203 (2)	0.4333 (3)	0.0858 (17)	
H47A	2.719630	−0.015909	0.411752	0.129*	
H47B	2.756371	0.050690	0.400056	0.129*	
H47C	2.833672	0.012977	0.457141	0.129*	
C48	3.3194 (9)	0.4272 (3)	0.5651 (4)	0.226 (6)	
N1	2.7024 (3)	0.18535 (14)	0.60652 (16)	0.0451 (8)	
N2	2.6641 (3)	0.13278 (15)	0.57286 (17)	0.0515 (8)	
H2A	2.612437	0.108611	0.582327	0.077*	
N3	3.5183 (3)	0.05500 (16)	0.68568 (19)	0.0572 (9)	
N4	2.6876 (4)	0.3986 (2)	0.8257 (2)	0.0806 (12)	
O1	3.6175 (3)	0.07496 (16)	0.71350 (18)	0.0825 (10)	
O2	3.4847 (3)	0.06823 (18)	0.62581 (19)	0.0957 (13)	
O3	3.4555 (4)	0.02164 (18)	0.71447 (19)	0.0932 (12)	
P1	3.04707 (8)	0.20709 (4)	0.63954 (5)	0.0408 (2)	
P2	2.80883 (9)	0.30789 (4)	0.51150 (5)	0.0408 (3)	
S1	2.84891 (10)	0.14034 (5)	0.49953 (5)	0.0546 (3)	
S2	2.65109 (13)	0.04460 (6)	0.49112 (7)	0.0821 (4)	
Cu	2.86077 (4)	0.22336 (2)	0.57262 (2)	0.04075 (17)	
Cl1	3.2286 (4)	0.39592 (19)	0.4997 (3)	0.1015 (9)	0.5
Cl1'	3.4001 (3)	0.3864 (2)	0.6323 (2)	0.1015 (9)	0.5
Cl2	3.4040 (14)	0.4819 (4)	0.5416 (7)	0.360 (7)	0.5
Cl2'	3.3799 (15)	0.3925 (4)	0.6356 (6)	0.360 (7)	0.5
Cl3	3.2687 (9)	0.4928 (3)	0.5874 (4)	0.215 (3)	0.5
Cl3'	3.2528 (9)	0.3816 (3)	0.5083 (4)	0.215 (3)	0.5
Cl4	3.2245 (9)	0.4611 (8)	0.6087 (8)	0.380 (7)	0.5
Cl4'	3.4426 (10)	0.4662 (7)	0.5482 (10)	0.380 (7)	0.5

### {Methyl (Z)-2-[4-(dimethylamino)benzylidene]hydrazine-1-carbodithioate-κ2N2,S}bis(triphenylphosphine-κP)copper(I) nitrate carbon tetrachloride monosolvate Atomic displacement parameters (Å^2^)

**Table:**

	*U*^11^	*U*^22^	*U*^33^	*U*^12^	*U*^13^	*U*^23^
C1	0.051 (2)	0.049 (2)	0.040 (2)	−0.0006 (18)	0.0054 (16)	0.0050 (17)
C2	0.051 (2)	0.060 (3)	0.055 (2)	0.000 (2)	0.0083 (19)	0.013 (2)
C3	0.069 (3)	0.077 (3)	0.072 (3)	−0.011 (3)	0.011 (2)	0.029 (3)
C4	0.083 (3)	0.070 (3)	0.082 (4)	−0.003 (3)	0.005 (3)	0.036 (3)
C5	0.070 (3)	0.070 (3)	0.084 (3)	0.014 (3)	0.001 (3)	0.026 (3)
C6	0.053 (2)	0.066 (3)	0.070 (3)	0.005 (2)	0.010 (2)	0.017 (2)
C7	0.0407 (19)	0.046 (2)	0.040 (2)	0.0007 (16)	0.0028 (15)	0.0044 (17)
C8	0.058 (2)	0.059 (3)	0.063 (3)	−0.006 (2)	0.016 (2)	−0.002 (2)
C9	0.069 (3)	0.069 (3)	0.066 (3)	0.011 (2)	0.025 (2)	−0.007 (2)
C10	0.061 (3)	0.095 (4)	0.060 (3)	0.011 (3)	0.025 (2)	0.005 (3)
C11	0.054 (3)	0.086 (4)	0.074 (3)	−0.015 (2)	0.020 (2)	0.011 (3)
C12	0.053 (2)	0.059 (3)	0.059 (3)	−0.008 (2)	0.012 (2)	−0.001 (2)
C13	0.0402 (19)	0.061 (3)	0.045 (2)	−0.0083 (18)	0.0096 (17)	−0.0076 (19)
C14	0.057 (2)	0.082 (3)	0.048 (2)	−0.016 (2)	0.001 (2)	−0.001 (2)
C15	0.076 (3)	0.129 (5)	0.052 (3)	−0.033 (4)	0.002 (2)	−0.015 (3)
C16	0.088 (4)	0.131 (6)	0.077 (4)	−0.050 (4)	0.016 (3)	−0.054 (4)
C17	0.089 (4)	0.073 (4)	0.113 (5)	−0.019 (3)	0.024 (4)	−0.037 (3)
C18	0.059 (3)	0.057 (3)	0.078 (3)	−0.010 (2)	0.001 (2)	−0.016 (2)
C19	0.048 (2)	0.042 (2)	0.042 (2)	−0.0087 (17)	0.0058 (16)	0.0003 (17)
C20	0.054 (2)	0.074 (3)	0.055 (3)	0.005 (2)	0.009 (2)	0.015 (2)
C21	0.063 (3)	0.095 (4)	0.068 (3)	0.008 (3)	0.021 (2)	0.009 (3)
C22	0.070 (3)	0.095 (4)	0.054 (3)	−0.012 (3)	0.023 (2)	0.005 (3)
C23	0.078 (3)	0.069 (3)	0.052 (3)	−0.012 (3)	0.013 (2)	0.017 (2)
C24	0.066 (3)	0.057 (3)	0.055 (3)	0.003 (2)	0.012 (2)	0.016 (2)
C25	0.063 (2)	0.039 (2)	0.044 (2)	0.0020 (18)	0.0031 (19)	0.0011 (17)
C26	0.085 (3)	0.057 (3)	0.075 (3)	−0.014 (2)	0.017 (3)	−0.012 (2)
C27	0.116 (5)	0.061 (4)	0.110 (5)	−0.030 (3)	0.009 (4)	−0.021 (3)
C28	0.155 (6)	0.056 (4)	0.078 (4)	0.001 (4)	−0.001 (4)	−0.018 (3)
C29	0.127 (5)	0.066 (4)	0.062 (3)	0.017 (3)	0.027 (3)	−0.004 (3)
C30	0.083 (3)	0.052 (3)	0.055 (3)	0.006 (2)	0.018 (2)	−0.001 (2)
C31	0.042 (2)	0.060 (3)	0.039 (2)	0.0004 (18)	0.0079 (16)	0.0065 (19)
C32	0.078 (3)	0.076 (4)	0.074 (3)	0.018 (3)	−0.009 (3)	0.005 (3)
C33	0.072 (3)	0.123 (5)	0.083 (4)	0.035 (3)	−0.009 (3)	0.022 (4)
C34	0.049 (3)	0.139 (6)	0.059 (3)	−0.002 (3)	−0.001 (2)	0.012 (3)
C35	0.064 (3)	0.113 (5)	0.058 (3)	−0.027 (3)	0.000 (2)	−0.005 (3)
C36	0.054 (2)	0.070 (3)	0.052 (2)	−0.013 (2)	0.0005 (19)	0.000 (2)
C37	0.044 (2)	0.050 (2)	0.057 (2)	−0.0090 (18)	0.0055 (19)	0.013 (2)
C38	0.047 (2)	0.052 (2)	0.050 (2)	0.0038 (19)	0.0126 (18)	0.012 (2)
C39	0.047 (2)	0.063 (3)	0.054 (2)	−0.0023 (19)	0.0130 (19)	−0.002 (2)
C40	0.058 (3)	0.067 (3)	0.053 (3)	−0.008 (2)	0.010 (2)	−0.001 (2)
C41	0.077 (3)	0.059 (3)	0.051 (3)	0.008 (2)	0.018 (2)	0.009 (2)
C42	0.064 (3)	0.081 (4)	0.076 (3)	0.011 (3)	0.026 (2)	−0.004 (3)
C43	0.048 (2)	0.081 (4)	0.080 (3)	−0.004 (2)	0.021 (2)	−0.001 (3)
C44	0.144 (6)	0.082 (4)	0.074 (4)	−0.021 (4)	0.029 (4)	−0.016 (3)
C45	0.116 (5)	0.107 (5)	0.079 (4)	0.029 (4)	0.020 (3)	−0.019 (3)
C46	0.058 (2)	0.039 (2)	0.053 (2)	−0.0109 (18)	−0.012 (2)	0.0025 (18)
C47	0.134 (5)	0.054 (3)	0.069 (3)	−0.031 (3)	0.013 (3)	−0.015 (2)
C48	0.276 (14)	0.118 (8)	0.269 (14)	−0.057 (9)	−0.022 (12)	−0.039 (9)
N1	0.0427 (17)	0.0447 (19)	0.0464 (18)	−0.0082 (14)	0.0007 (14)	0.0075 (15)
N2	0.0520 (19)	0.045 (2)	0.056 (2)	−0.0173 (15)	0.0019 (16)	0.0032 (16)
N3	0.058 (2)	0.054 (2)	0.061 (2)	−0.0015 (18)	0.0158 (19)	0.0054 (18)
N4	0.100 (3)	0.074 (3)	0.073 (3)	−0.001 (2)	0.030 (2)	−0.013 (2)
O1	0.078 (2)	0.088 (3)	0.078 (2)	−0.0154 (19)	−0.0019 (18)	0.0061 (19)
O2	0.091 (2)	0.112 (3)	0.077 (2)	−0.051 (2)	−0.013 (2)	0.030 (2)
O3	0.091 (2)	0.104 (3)	0.090 (3)	−0.022 (2)	0.032 (2)	0.021 (2)
P1	0.0396 (5)	0.0427 (6)	0.0395 (5)	−0.0033 (4)	0.0035 (4)	0.0016 (4)
P2	0.0437 (5)	0.0386 (6)	0.0397 (5)	−0.0037 (4)	0.0044 (4)	0.0024 (4)
S1	0.0611 (6)	0.0450 (6)	0.0580 (6)	−0.0121 (5)	0.0096 (5)	−0.0105 (5)
S2	0.0927 (9)	0.0565 (8)	0.0950 (10)	−0.0351 (7)	0.0060 (8)	−0.0151 (7)
Cu	0.0427 (3)	0.0374 (3)	0.0413 (3)	−0.00711 (19)	0.00314 (19)	0.00170 (19)
Cl1	0.0810 (13)	0.091 (2)	0.125 (2)	−0.0002 (12)	−0.0107 (13)	−0.0179 (16)
Cl1'	0.0810 (13)	0.091 (2)	0.125 (2)	−0.0002 (12)	−0.0107 (13)	−0.0179 (16)
Cl2	0.654 (18)	0.123 (4)	0.265 (8)	−0.131 (7)	−0.071 (9)	0.054 (5)
Cl2'	0.654 (18)	0.123 (4)	0.265 (8)	−0.131 (7)	−0.071 (9)	0.054 (5)
Cl3	0.307 (7)	0.114 (3)	0.190 (4)	0.064 (3)	−0.088 (4)	−0.027 (3)
Cl3'	0.307 (7)	0.114 (3)	0.190 (4)	0.064 (3)	−0.088 (4)	−0.027 (3)
Cl4	0.179 (5)	0.483 (15)	0.499 (16)	−0.046 (7)	0.113 (7)	−0.183 (13)
Cl4'	0.179 (5)	0.483 (15)	0.499 (16)	−0.046 (7)	0.113 (7)	−0.183 (13)

### {Methyl (Z)-2-[4-(dimethylamino)benzylidene]hydrazine-1-carbodithioate-κ2N2,S}bis(triphenylphosphine-κP)copper(I) nitrate carbon tetrachloride monosolvate Geometric parameters (Å, º)

**Table:**

C1—C6	1.391 (6)	C30—H30	0.9300
C1—C2	1.392 (5)	C31—C32	1.367 (6)
C1—P1	1.821 (4)	C31—C36	1.387 (6)
C2—C3	1.386 (6)	C31—P2	1.840 (4)
C2—H2	0.9300	C32—C33	1.395 (7)
C3—C4	1.370 (7)	C32—H32	0.9300
C3—H3	0.9300	C33—C34	1.364 (8)
C4—C5	1.356 (7)	C33—H33	0.9300
C4—H4	0.9300	C34—C35	1.338 (8)
C5—C6	1.390 (6)	C34—H34	0.9300
C5—H5	0.9300	C35—C36	1.395 (6)
C6—H6	0.9300	C35—H35	0.9300
C7—C12	1.360 (6)	C36—H36	0.9300
C7—C8	1.404 (6)	C37—N1	1.287 (5)
C7—P1	1.830 (4)	C37—C38	1.440 (6)
C8—C9	1.382 (6)	C37—H37	0.9300
C8—H8	0.9300	C38—C39	1.386 (5)
C9—C10	1.365 (7)	C38—C43	1.398 (6)
C9—H9	0.9300	C39—C40	1.360 (6)
C10—C11	1.369 (7)	C39—H39	0.9300
C10—H10	0.9300	C40—C41	1.400 (6)
C11—C12	1.405 (6)	C40—H40	0.9300
C11—H11	0.9300	C41—N4	1.372 (6)
C12—H12	0.9300	C41—C42	1.395 (7)
C13—C18	1.379 (6)	C42—C43	1.360 (7)
C13—C14	1.392 (5)	C42—H42	0.9300
C13—P1	1.821 (4)	C43—H43	0.9300
C14—C15	1.352 (7)	C44—N4	1.419 (7)
C14—H14	0.9300	C44—H44A	0.9600
C15—C16	1.359 (9)	C44—H44B	0.9600
C15—H15	0.9300	C44—H44C	0.9600
C16—C17	1.378 (9)	C45—N4	1.438 (7)
C16—H16	0.9300	C45—H45A	0.9600
C17—C18	1.393 (7)	C45—H45B	0.9600
C17—H17	0.9300	C45—H45C	0.9600
C18—H18	0.9300	C46—N2	1.320 (5)
C19—C20	1.378 (6)	C46—S1	1.668 (4)
C19—C24	1.386 (5)	C46—S2	1.759 (4)
C19—P2	1.827 (4)	C47—S2	1.788 (6)
C20—C21	1.395 (6)	C47—H47A	0.9600
C20—H20	0.9300	C47—H47B	0.9600
C21—C22	1.370 (7)	C47—H47C	0.9600
C21—H21	0.9300	C48—Cl3'	1.622 (8)
C22—C23	1.362 (7)	C48—Cl4	1.630 (10)
C22—H22	0.9300	C48—Cl2	1.639 (11)
C23—C24	1.384 (6)	C48—Cl3	1.652 (10)
C23—H23	0.9300	C48—Cl2'	1.665 (10)
C24—H24	0.9300	C48—Cl4'	1.670 (10)
C25—C30	1.376 (6)	C48—Cl1	1.680 (8)
C25—C26	1.384 (6)	C48—Cl1'	1.757 (9)
C25—P2	1.821 (4)	N1—N2	1.390 (4)
C26—C27	1.375 (7)	N1—Cu	2.112 (3)
C26—H26	0.9300	N2—H2A	0.8200
C27—C28	1.380 (9)	N3—O3	1.211 (5)
C27—H27	0.9300	N3—O1	1.223 (4)
C28—C29	1.365 (8)	N3—O2	1.243 (5)
C28—H28	0.9300	P1—Cu	2.2911 (10)
C29—C30	1.380 (7)	P2—Cu	2.2808 (11)
C29—H29	0.9300	S1—Cu	2.3599 (11)
			
C6—C1—C2	118.3 (4)	C31—C32—C33	119.0 (5)
C6—C1—P1	123.8 (3)	C31—C32—H32	120.5
C2—C1—P1	117.9 (3)	C33—C32—H32	120.5
C3—C2—C1	121.1 (4)	C34—C33—C32	121.5 (5)
C3—C2—H2	119.5	C34—C33—H33	119.3
C1—C2—H2	119.5	C32—C33—H33	119.3
C4—C3—C2	119.2 (4)	C35—C34—C33	119.0 (5)
C4—C3—H3	120.4	C35—C34—H34	120.5
C2—C3—H3	120.4	C33—C34—H34	120.5
C5—C4—C3	121.0 (4)	C34—C35—C36	121.7 (5)
C5—C4—H4	119.5	C34—C35—H35	119.1
C3—C4—H4	119.5	C36—C35—H35	119.1
C4—C5—C6	120.5 (4)	C31—C36—C35	118.9 (5)
C4—C5—H5	119.8	C31—C36—H36	120.5
C6—C5—H5	119.8	C35—C36—H36	120.5
C5—C6—C1	120.0 (4)	N1—C37—C38	125.9 (4)
C5—C6—H6	120.0	N1—C37—H37	117.1
C1—C6—H6	120.0	C38—C37—H37	117.1
C12—C7—C8	118.5 (4)	C39—C38—C43	115.9 (4)
C12—C7—P1	122.6 (3)	C39—C38—C37	124.4 (4)
C8—C7—P1	118.9 (3)	C43—C38—C37	119.6 (4)
C9—C8—C7	120.8 (4)	C40—C39—C38	122.2 (4)
C9—C8—H8	119.6	C40—C39—H39	118.9
C7—C8—H8	119.6	C38—C39—H39	118.9
C10—C9—C8	119.8 (5)	C39—C40—C41	121.7 (4)
C10—C9—H9	120.1	C39—C40—H40	119.2
C8—C9—H9	120.1	C41—C40—H40	119.2
C9—C10—C11	120.3 (4)	N4—C41—C42	121.6 (4)
C9—C10—H10	119.8	N4—C41—C40	121.9 (5)
C11—C10—H10	119.8	C42—C41—C40	116.5 (4)
C10—C11—C12	120.0 (4)	C43—C42—C41	121.2 (4)
C10—C11—H11	120.0	C43—C42—H42	119.4
C12—C11—H11	120.0	C41—C42—H42	119.4
C7—C12—C11	120.6 (4)	C42—C43—C38	122.5 (4)
C7—C12—H12	119.7	C42—C43—H43	118.8
C11—C12—H12	119.7	C38—C43—H43	118.8
C18—C13—C14	118.0 (4)	N4—C44—H44A	109.5
C18—C13—P1	118.7 (3)	N4—C44—H44B	109.5
C14—C13—P1	123.3 (4)	H44A—C44—H44B	109.5
C15—C14—C13	121.3 (5)	N4—C44—H44C	109.5
C15—C14—H14	119.3	H44A—C44—H44C	109.5
C13—C14—H14	119.3	H44B—C44—H44C	109.5
C14—C15—C16	120.2 (5)	N4—C45—H45A	109.5
C14—C15—H15	119.9	N4—C45—H45B	109.5
C16—C15—H15	119.9	H45A—C45—H45B	109.5
C15—C16—C17	120.9 (5)	N4—C45—H45C	109.5
C15—C16—H16	119.5	H45A—C45—H45C	109.5
C17—C16—H16	119.5	H45B—C45—H45C	109.5
C16—C17—C18	118.7 (6)	N2—C46—S1	125.0 (3)
C16—C17—H17	120.7	N2—C46—S2	111.6 (3)
C18—C17—H17	120.7	S1—C46—S2	123.4 (3)
C13—C18—C17	120.8 (5)	S2—C47—H47A	109.5
C13—C18—H18	119.6	S2—C47—H47B	109.5
C17—C18—H18	119.6	H47A—C47—H47B	109.5
C20—C19—C24	118.2 (4)	S2—C47—H47C	109.5
C20—C19—P2	119.4 (3)	H47A—C47—H47C	109.5
C24—C19—P2	122.4 (3)	H47B—C47—H47C	109.5
C19—C20—C21	121.1 (4)	Cl3'—C48—Cl3	128.2 (7)
C19—C20—H20	119.4	Cl2—C48—Cl2'	115.1 (8)
C21—C20—H20	119.4	Cl4—C48—Cl4'	116.8 (9)
C22—C21—C20	119.4 (5)	Cl1—C48—Cl1'	124.0 (5)
C22—C21—H21	120.3	C37—N1—N2	113.1 (3)
C20—C21—H21	120.3	C37—N1—Cu	134.0 (3)
C23—C22—C21	120.1 (4)	N2—N1—Cu	112.5 (2)
C23—C22—H22	119.9	C46—N2—N1	122.1 (3)
C21—C22—H22	119.9	C46—N2—H2A	109.5
C22—C23—C24	120.6 (4)	N1—N2—H2A	127.7
C22—C23—H23	119.7	O3—N3—O1	121.5 (4)
C24—C23—H23	119.7	O3—N3—O2	120.1 (4)
C23—C24—C19	120.4 (4)	O1—N3—O2	118.3 (4)
C23—C24—H24	119.8	C41—N4—C44	121.1 (4)
C19—C24—H24	119.8	C41—N4—C45	119.7 (5)
C30—C25—C26	117.4 (4)	C44—N4—C45	119.2 (5)
C30—C25—P2	119.5 (3)	C13—P1—C1	105.10 (18)
C26—C25—P2	122.9 (3)	C13—P1—C7	102.20 (17)
C27—C26—C25	121.6 (5)	C1—P1—C7	103.01 (18)
C27—C26—H26	119.2	C13—P1—Cu	119.84 (14)
C25—C26—H26	119.2	C1—P1—Cu	113.40 (12)
C26—C27—C28	120.0 (6)	C7—P1—Cu	111.46 (12)
C26—C27—H27	120.0	C25—P2—C19	104.41 (18)
C28—C27—H27	120.0	C25—P2—C31	103.36 (18)
C29—C28—C27	119.1 (5)	C19—P2—C31	100.75 (17)
C29—C28—H28	120.4	C25—P2—Cu	118.12 (13)
C27—C28—H28	120.4	C19—P2—Cu	111.82 (13)
C28—C29—C30	120.6 (5)	C31—P2—Cu	116.33 (13)
C28—C29—H29	119.7	C46—S1—Cu	95.54 (16)
C30—C29—H29	119.7	C46—S2—C47	103.4 (2)
C25—C30—C29	121.4 (5)	N1—Cu—P2	110.95 (9)
C25—C30—H30	119.3	N1—Cu—P1	115.78 (8)
C29—C30—H30	119.3	P2—Cu—P1	125.29 (4)
C32—C31—C36	119.8 (4)	N1—Cu—S1	84.80 (10)
C32—C31—P2	123.3 (3)	P2—Cu—S1	109.19 (4)
C36—C31—P2	116.5 (3)	P1—Cu—S1	101.91 (4)
			
C6—C1—C2—C3	1.2 (6)	N4—C41—C42—C43	178.9 (5)
P1—C1—C2—C3	179.8 (4)	C40—C41—C42—C43	−1.3 (7)
C1—C2—C3—C4	−0.1 (7)	C41—C42—C43—C38	−0.9 (8)
C2—C3—C4—C5	−0.5 (8)	C39—C38—C43—C42	2.4 (7)
C3—C4—C5—C6	−0.1 (9)	C37—C38—C43—C42	−179.2 (4)
C4—C5—C6—C1	1.2 (8)	C38—C37—N1—N2	174.1 (3)
C2—C1—C6—C5	−1.8 (7)	C38—C37—N1—Cu	−13.9 (6)
P1—C1—C6—C5	179.7 (4)	S1—C46—N2—N1	0.1 (5)
C12—C7—C8—C9	1.3 (6)	S2—C46—N2—N1	−178.8 (2)
P1—C7—C8—C9	177.8 (3)	C37—N1—N2—C46	174.2 (3)
C7—C8—C9—C10	−1.7 (7)	Cu—N1—N2—C46	0.4 (4)
C8—C9—C10—C11	0.4 (7)	C42—C41—N4—C44	−177.2 (5)
C9—C10—C11—C12	1.2 (7)	C40—C41—N4—C44	3.0 (7)
C8—C7—C12—C11	0.2 (6)	C42—C41—N4—C45	6.2 (7)
P1—C7—C12—C11	−176.1 (3)	C40—C41—N4—C45	−173.5 (5)
C10—C11—C12—C7	−1.5 (7)	C18—C13—P1—C1	−150.4 (3)
C18—C13—C14—C15	−1.1 (7)	C14—C13—P1—C1	30.4 (4)
P1—C13—C14—C15	178.0 (4)	C18—C13—P1—C7	102.3 (4)
C13—C14—C15—C16	1.3 (8)	C14—C13—P1—C7	−76.8 (4)
C14—C15—C16—C17	−0.9 (9)	C18—C13—P1—Cu	−21.5 (4)
C15—C16—C17—C18	0.4 (9)	C14—C13—P1—Cu	159.4 (3)
C14—C13—C18—C17	0.6 (7)	C6—C1—P1—C13	−79.5 (4)
P1—C13—C18—C17	−178.6 (4)	C2—C1—P1—C13	101.9 (3)
C16—C17—C18—C13	−0.3 (8)	C6—C1—P1—C7	27.2 (4)
C24—C19—C20—C21	1.4 (6)	C2—C1—P1—C7	−151.4 (3)
P2—C19—C20—C21	−179.5 (4)	C6—C1—P1—Cu	147.8 (3)
C19—C20—C21—C22	1.3 (7)	C2—C1—P1—Cu	−30.8 (4)
C20—C21—C22—C23	−2.5 (8)	C12—C7—P1—C13	−24.3 (4)
C21—C22—C23—C24	1.0 (8)	C8—C7—P1—C13	159.3 (3)
C22—C23—C24—C19	1.7 (7)	C12—C7—P1—C1	−133.2 (3)
C20—C19—C24—C23	−2.9 (6)	C8—C7—P1—C1	50.5 (3)
P2—C19—C24—C23	178.0 (3)	C12—C7—P1—Cu	104.9 (3)
C30—C25—C26—C27	0.9 (7)	C8—C7—P1—Cu	−71.5 (3)
P2—C25—C26—C27	−174.5 (4)	C30—C25—P2—C19	165.8 (3)
C25—C26—C27—C28	−1.2 (9)	C26—C25—P2—C19	−19.0 (4)
C26—C27—C28—C29	0.7 (10)	C30—C25—P2—C31	60.8 (4)
C27—C28—C29—C30	0.2 (9)	C26—C25—P2—C31	−124.0 (4)
C26—C25—C30—C29	0.0 (7)	C30—C25—P2—Cu	−69.3 (4)
P2—C25—C30—C29	175.5 (4)	C26—C25—P2—Cu	106.0 (4)
C28—C29—C30—C25	−0.5 (8)	C20—C19—P2—C25	117.1 (3)
C36—C31—C32—C33	−1.3 (7)	C24—C19—P2—C25	−63.8 (4)
P2—C31—C32—C33	171.9 (4)	C20—C19—P2—C31	−135.9 (3)
C31—C32—C33—C34	−0.3 (9)	C24—C19—P2—C31	43.2 (4)
C32—C33—C34—C35	1.7 (9)	C20—C19—P2—Cu	−11.7 (4)
C33—C34—C35—C36	−1.5 (8)	C24—C19—P2—Cu	167.4 (3)
C32—C31—C36—C35	1.5 (6)	C32—C31—P2—C25	17.6 (4)
P2—C31—C36—C35	−172.1 (3)	C36—C31—P2—C25	−169.0 (3)
C34—C35—C36—C31	−0.1 (7)	C32—C31—P2—C19	−90.1 (4)
N1—C37—C38—C39	−15.1 (6)	C36—C31—P2—C19	83.2 (3)
N1—C37—C38—C43	166.7 (4)	C32—C31—P2—Cu	148.8 (4)
C43—C38—C39—C40	−1.6 (6)	C36—C31—P2—Cu	−37.8 (3)
C37—C38—C39—C40	−179.9 (4)	N2—C46—S1—Cu	−0.5 (4)
C38—C39—C40—C41	−0.6 (7)	S2—C46—S1—Cu	178.3 (2)
C39—C40—C41—N4	−178.1 (4)	N2—C46—S2—C47	−176.3 (3)
C39—C40—C41—C42	2.1 (7)	S1—C46—S2—C47	4.8 (3)

### {Methyl (Z)-2-[4-(dimethylamino)benzylidene]hydrazine-1-carbodithioate-κ2N2,S}bis(triphenylphosphine-κP)copper(I) nitrate carbon tetrachloride monosolvate Hydrogen-bond geometry (Å, º)

**Table:**

*D*—H···*A*	*D*—H	H···*A*	*D*···*A*	*D*—H···*A*
N2—H2*A*···O2^i^	0.82	1.96	2.754 (5)	163
C2—H2···N1	0.93	2.55	3.391 (5)	151
C36—H36···S1	0.93	2.87	3.758 (5)	160
C37—H37···O2^i^	0.93	2.66	3.384 (5)	135
C47—H47*A*···O2	0.96	2.52	3.322 (6)	141

Symmetry code: (i) *x*−1, *y*, *z*.
